# Alternative B Cell Differentiation During Infection and Inflammation

**DOI:** 10.3389/fimmu.2022.908034

**Published:** 2022-06-24

**Authors:** Alan-Dine Courey-Ghaouzi, Linn Kleberg, Christopher Sundling

**Affiliations:** ^1^Division of Infectious Diseases, Department of Medicine Solna and Center for Molecular Medicine, Karolinska Institutet, Stockholm, Sweden; ^2^Department of Infectious Diseases, Karolinska University Hospital, Stockholm, Sweden

**Keywords:** atypical memory B cells, tissue-like B cells, double-negative B cells, T-bet, CD11c, FcRL5, CD21

## Abstract

Long-term protective immunity to infectious disease depends on cell-mediated and humoral immune responses. Induction of a strong humoral response relies on efficient B cell activation and differentiation to long-lived plasma cells and memory B cells. For many viral or bacterial infections, a single encounter is sufficient to induce such responses. In malaria, the induction of long-term immunity can take years of pathogen exposure to develop, if it occurs at all. This repeated pathogen exposure and suboptimal immune response coincide with the expansion of a subset of B cells, often termed atypical memory B cells. This subset is present at low levels in healthy individuals as well but it is observed to expand in an inflammatory context during acute and chronic infection, autoimmune diseases or certain immunodeficiencies. Therefore, it has been proposed that this subset is exhausted, dysfunctional, or potentially autoreactive, but its actual role has remained elusive. Recent reports have provided new information regarding both heterogeneity and expansion of these cells, in addition to indications on their potential role during normal immune responses to infection or vaccination. These new insights encourage us to rethink how and why they are generated and better understand their role in our complex immune system. In this review, we will focus on recent advances in our understanding of these enigmatic cells and highlight the remaining gaps that need to be filled.

## Introduction

Vaccines constitute one of the most successful medical inventions known to date and have allowed the eradication or control of several previously common and deadly diseases. However, despite considerable efforts, vaccine development has proven difficult for some infections. For many of the vaccines, the best correlate of protection is humoral immune responses, derived from long-lived B cell memory, in the form of antibody-secreting plasma cells (ASCs) and memory B cells (MBCs) (1).

Long-lived B cell responses are generated following antigen encounter by naïve B cells and subsequent interactions with activated cognate CD4 helper T (Th) cells at the T-B border in secondary lymphoid organs (2). This initial extrafollicular interaction promotes B cell receptor (BCR) class-switching (3) and differentiation to either of several fates, such as early memory B cells, short-lived antibody-secreting cells (ASCs), or germinal center (GC) B cells in an antigen and affinity-dependent manner (4–6). B cells fated for GC selection enter the B cell follicles where they start to rapidly proliferate and form a dark zone and light zone GC structure (7). Within the GC, the B cells will undergo isotype-dependent selection (8) and affinity maturation to eventually differentiate to long-lived MBCs and ASCs (9–12). The MBCs will then circulate between secondary lymphoid organs *via* the blood, or reside at sites of infection or inflammation (13), while the ASCs establish themselves in specialized niches that can sustain their extensive antibody production (14).

In addition to antibody production, B cells also have important roles as regulators of the immune response. Both by secreting inflammatory mediators, such as TNF-α and IL-6 (15), driving inflammation but also suppressing excessive inflammation through secretion of IL-10 and metabolizing extracellular ATP to ADP (16–18). Additionally, B cells are professional antigen-presenting cells (APCs) as they express high levels of MHC class II and can rapidly upregulate co-stimulatory molecules, such as CD80, CD86, and ICOSL upon stimulation. This interaction is important for subsequent B cell responses but also to drive T follicular helper (Tfh) cell differentiation at the B cell-T cell interfollicular region (19) and is proposed to promote effector T cell responses at sites of inflammation (20, 21).

In addition to the classical cell fates, B cells have also been shown to differentiate to an alternative B cell subset, often denoted as atypical, pro-inflammatory, exhausted, CD27^–^IgD^lo^ double negative, or tissue-like B cells, depending on the context in which they were identified (22). In this minireview, we further describe these cells in the context of different diseases or vaccination and highlight what is known about their origin, differentiation, migration and what potential function they might have within the immune response. As the nomenclature of these cells varies greatly between different studies, we have strived to use the most common overlapping markers used in the different papers.

## Alternative B Cell Differentiation in Disease and Vaccination

Resting naïve and memory B cells express complement receptor 2 (CD21), a co-receptor for the BCR (23), that is important in reducing the activation threshold upon BCR ligation (24). In 2002 Warnatz et al. described a B cell subset lacking CD21 expression in immunodeficient patients (25). Ehrhardt et al. reported in 2005 a similar B cell subset, lacking CD21 and the memory marker CD27 in different tissues and B cell lines (26). Although the markers used to distinguish this alternative B cell subset have not been firmly established, expression of the transcription factor T-bet, the integrin CD11c, different Fc receptor-like (FcRL) proteins, and chemokine receptors (such as CXCR3) have been used (27). This subset of B cells is present in healthy individuals at low levels (28) and increases with age (29), but also expands greatly during inflammatory conditions, including infections, autoimmune disorders, and after vaccination (30, 31).

In 2009 Weiss et al. reported the expansion of a subpopulation of B cells amongst people living in malaria-endemic areas (32). The B cells were referred to as atypical memory B cells and were identified through the low expression of CD21 and CD27 (32). Several additional studies have since shown that CD21^lo^CD27^lo^ B cells greatly expand during infection with malaria parasites (33–38). Tissue-like memory B cells, which have a similar CD21^lo^CD27^lo^ phenotype as the atypical memory B cells, but also express FcRL4 are described to expand in individuals infected with human immunodeficiency virus (HIV) (39–41). The cells showed reduced BCR signaling and antibody production upon stimulation, leading to the thought that the B cells were anergic or exhausted (39). Other infections where B cells with a CD21^lo^CD27^lo^IgD^−^ phenotype are observed to expand are hepatitis C virus (HCV) (42, 43), severe acute respiratory syndrome coronavirus 2 (SARS-CoV-2) (44–46), hantavirus infection (47) tuberculosis (48), and possibly others.

In the autoimmunity field, B cells lacking CD27 and IgD, often termed double negative (DN) B cells, are seen to increase in various diseases with inflammatory components. In systemic lupus erythematosus (SLE), the DN B cells express CD11c, FcRL5 and T-bet (49, 50), markers largely overlapping with those expressed during infection. Similar CD21^lo^CD27^lo^CD11c^+^ B cells have also been reported to expand in a subset of in patients with common variable immunodeficiency (CVID), rheumatoid arthritis (51) and multiple sclerosis (52). This phenotypic overlap is further supported by largely overlapping transcriptional programs based on bulk microarray and RNA sequencing of CD11c^hi^ cells (53) and single-cell RNA sequencing (54).

## Mechanisms of Alternative B Cell Differentiation

**Figure 1:**
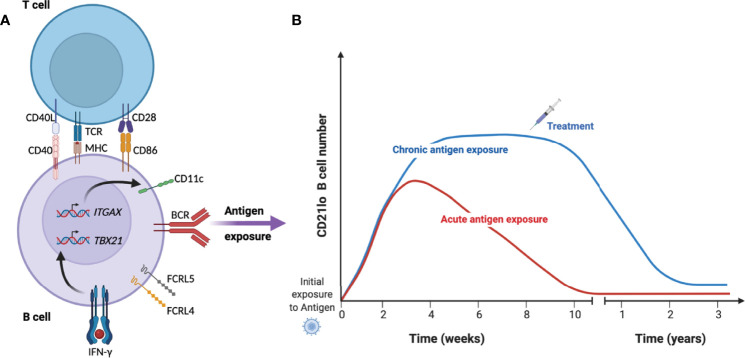
Kinetics of CD21^lo^ CD11c^+^ T-bet^+^ B cells during acute and chronic disease. **(A)** Under specific conditions such as B cell receptor (BCR) activation in the presence of IFN-γ and T cell help, B cell will acquire a phenotype characterized by reduced expression of CD21 and increased expression of CD11c (*ITGAX*), T-bet (*TBX21*) and FCRL4/5. **(B)** They then rapidly expand over several weeks until they start to contract over several months in the absence of antigen (red line). In the context of chronic infection or repeated infection (blue line), the cells can remain a substantial proportion of total B cells until treatment removes the source of antigen and reduce the inflammatory response.

Although initial observations of expanded CD21^lo^ B cell numbers were primarily in the context of chronic immune activation, more recent studies in both mice and humans have shown that CD21^lo^ T-bet^+^ or CD11c^+^ B cells expand rapidly after infection or immunization (36, 41, 45, 55), after which they slowly decline over several months (36, 38, 41, 56) (**Figure 1**). Similarly, these cells start to decline after treatment of individuals with HCV (43) and tuberculosis (48), indicating that the cells need a sustained proinflammatory environment and or antigen-stimulation to survive.

In malaria, the expansion of CD21^lo^CD27^lo^ B cells is associated with the intensity of parasite transmission (33), consistent with antigen exposure in a proinflammatory environment being important in driving expansion or survival of these cells (32, 57, 58). Studies have linked the expansion of T-bet^+^ B cells to the pro-inflammatory cytokine interferon-γ (IFN-γ), both in the context of malaria (59) and SLE (50, 60). IFN-γ is a T helper type 1 (Th1) cytokine, which upon binding to the IFN-γ receptor on B cells activates the JAK-STAT signaling pathway, resulting in up-regulation of the transcription factor T-bet (61), which is important for IgG2a/c class-switching in mice (62) and likely IgG3 in humans. In contrast to T-bet, upregulation of CD11c seems to require BCR-ligation but not IFN-γ (63), potentially explaining why these proteins are not always co-expressed. The differentiation to CD21^lo^ T-bet^+^ or CD11c^+^ B cells has primarily been described to start from classical MBCs (34, 64). However, many of the CD21^lo^ T-bet^+^ or CD11c^+^ B cells display an unswitched BCR (36), potentially indicating a naïve B cell origin. In support of this, BCR sequencing shows partly overlapping repertoire and gene characteristics between naïve B cells and unswitched CD21^lo^CD27^lo^ B cells (65, 66). Further, Obeng-Adjei et al. reported that Th1 and Tfh-1 cells could induce a T-bet^hi^ phenotype in naïve B cells after two days of co-culture together with the superantigen staphylococcal enterotoxin B (59).

Ambegaonkar et al. further investigated which stimuli lead to upregulation of T-bet in naïve, GC, or memory B cell subsets *in vitro* (67). They found that BCR-ligation together with IFN-γ and the TLR9-ligand CpG could effectively make naïve and memory B cells, but not GC B cells, upregulate T-bet in addition to other surface proteins associated with CD21^lo^ B cells. Interestingly, naïve B cells were more capable of upregulating T-bet compared with MBCs under the conditions tested (67). Keller et al. investigated the contribution of different signaling pathways important for the generation of CD21^lo^ cells *in vivo* by studying individuals with CVID (60). They observed that BCR ligation together with CD4^+^ T cell-derived CD40L, IFN-γ, and IL-21 are important for the expansion of CD21^lo^T-bet^hi^ B cells, thus proposing that the expansion of these cells *in vivo* is T cell-dependent (60). This is also consistent with recent studies in mice, where CD11c^+^T-bet^+^ B cells generated after viral or intracellular bacterial infection require interactions with Tfh cells (68, 69).

## Route of Differentiation

The more efficient upregulation of T-bet in naïve and memory B cells could indicate that CD11c^+^T-bet^+^ B cells are primarily generated *via* the extrafollicular route, rather than *via* the GC. Both extrafollicular and GC B cells can undergo class-switch recombination and somatic hypermutation (70, 71), although the extrafollicular response is more rapid and associated with an expansion of ASCs, further reviewed by Elsner and Schlomchik (72). Jenks et al. point to several features of CD11c^+^ DN2 B cells, found in SLE patients, associated with extrafollicular differentiation, such as the cells lacking expression of CXCR5, a chemokine receptor involved in migration to secondary lymphoid organs, and CD62L, important for the trafficking to lymph nodes (73). Such receptor expression has also been described for CD11c^+^ B cells during malaria and hepatitis infection (32, 74). Additionally, sequencing of BCRs showed a similar mutation level of IgG in DN2 cells and ASCs, but lower than for switched memory B cells, suggesting that the DN2 cells had not gone through the GC while sharing common developmental pathways with ASCs (73). However, in several other studies associated with infection or vaccination, the mutation level was similar between conventional memory B cells and CD11c^+^Tbet^+^ B cells (64, 75, 76). Drawing strong conclusions based on BCR sequence analysis can potentially be misleading since it is possible that the CD11c^+^T-bet^+^ cells originate from conventional MBCs that could have a GC origin (34, 64). Therefore, investigating unswitched CD11c^+^T-bet^+^ B cells with a likely naïve origin could provide more direct support for the extrafollicular route, as these cells are more unlikely to have entered a GC reaction.

Contrasting with an extrafollicular route of CD11c^+^T-bet^+^ B cell differentiation, several studies have identified T-bet^+^ B cells in ongoing GCs in mice after challenge with malaria parasites (77) or influenza virus (75). Similar to peripheral T-bet^+^ B cells, GC B cells also have reduced levels of CD21 but few studies present data on CD11c expression among GC B cells. CD21^lo^CD27^+^ B cells in human peripheral blood that also have reduced CXCR5 and CD62L and high expression of Fas, similar to the DN2 B cells, were suggested to have a GC origin by Lau et al. (55). This conclusion was, however, largely based on BCR sequence analysis and mutational evolution after vaccination.

Although it is attractive to say that CD11c^+^T-bet^+^ B cells only have one origin and differentiation route, the extrafollicular and GC pathways are not mutually exclusive. Furthermore, it is possible that the conditions of the inflammatory response largely determine the route. This has been further discussed by Elsner and Schlomchik (72), where they propose that high levels of IFN-γ suppresses Tfh development and subsequent GC responses, promoting differentiation *via* the extrafollicular route, while lower IFN-γ levels can allow for Tfh-mediated T-bet^+^ GC B cell differentiation. However, It remains difficult to formally prove which route the peripheral CD11c^+^T-bet^+^ B cells took upon differentiation, especially in humans, where fate mapping approaches are not possible.

## Association With Antibody-Secreting Cells

Using RNA sequencing, Wang et al. noted that CD11c^hi^ B cells in SLE had upregulated genes associated with ASC differentiation, such as *PRDM1* (Blimp-1), *AICDA* (AID), *XBP1, BMP6*, *EMP3*, and *S100A4* (49). Furthermore, after culturing CD11c^hi^ B cells together with anti-CD3-activated T cells for 11 days, 70% of the cells expressed a CD27^+^CD38^hi^ ASC phenotype (49). Consistent with this, Golinski et al. also found that a larger proportion of CD11c^+^ B cells differentiated into ASCs compared to CD11c^–^ B cells, in addition to secreting more IgM and IgG after 7 days of culture in the presence of BCR ligation, TLR9-ligand, and IL-21 (63). These observations contrast with previous reports on restimulation of CD21^lo^ B cells in malaria, HIV, and Hepatitis B, where the cells displayed reduced differentiation to ASCs compared with classical memory B cells (34, 35, 39). This could potentially be due to intrinsic differences in the cells associated with autoimmune versus infectious diseases. But it could also be associated with the experimental conditions and the cell types included in the analysis, where a CD11c sort would likely include more CD21^+^ resting memory B cells than the sort for CD21^lo^CD27^lo^ B cells. However, consistent with the CD11c^hi^ transcriptomic data (49), Hopp et al. also found the ASC-associated genes *PRDM1* and *CD38* upregulated in CD21^lo^CD27^lo^ B cells during acute malaria (78). Furthermore, restimulation of sorted CD21^lo^CD27^lo^ B cells with superantigen activated Tfh cells led to ASC differentiation with upregulation of CD38 and production of IgG and IgM antibodies (78). This indicates that these cells can differentiate to ASC although the process could be context-dependent, such as the availability of Tfh cells.

## Potential Functions of CD21^lo^ B Cells

Although CD21^lo^ B cells can represent up to 50% of the circulating B cells in people living in malaria-endemic areas (32–34, 79) and are generated rapidly after vaccination or infection and have been proposed to be a normal part of the immune response (80, 81), the potential function of these cells remains largely unclear. Based on increased cell surface expression of several inhibitory receptors, such as CD22, CD85j, and FcγRIIB, and reduced responsiveness to restimulation of sorted human CD21^lo^ FcRL5^+^ or FcRL4^+^ B cells, these cells have been hypothesized to be exhausted or dysfunctional (34, 35, 39). Muellenbeck et al. showed that the cells were enriched for self- or polyreactive BCR specificities (76), potentially indicating that they could have been made anergic to protect the host from autoimmunity. However, Muellenbeck et al. also found BCR specificities of the cells overlapping with antibodies in plasma and mRNA transcripts corresponding to secretory antibodies, suggesting that the cells could contribute to the circulating antibody pool (76). This potential role has been further substantiated by findings that T-bet^+^ B cells can produce antibodies binding to phosphatidylserine on red blood cells, possibly contributing to anemia during malaria (82, 83), and similarly able to produce self-reactive antibodies in human and mouse models of SLE (73, 80, 81).

**Figure 2:**
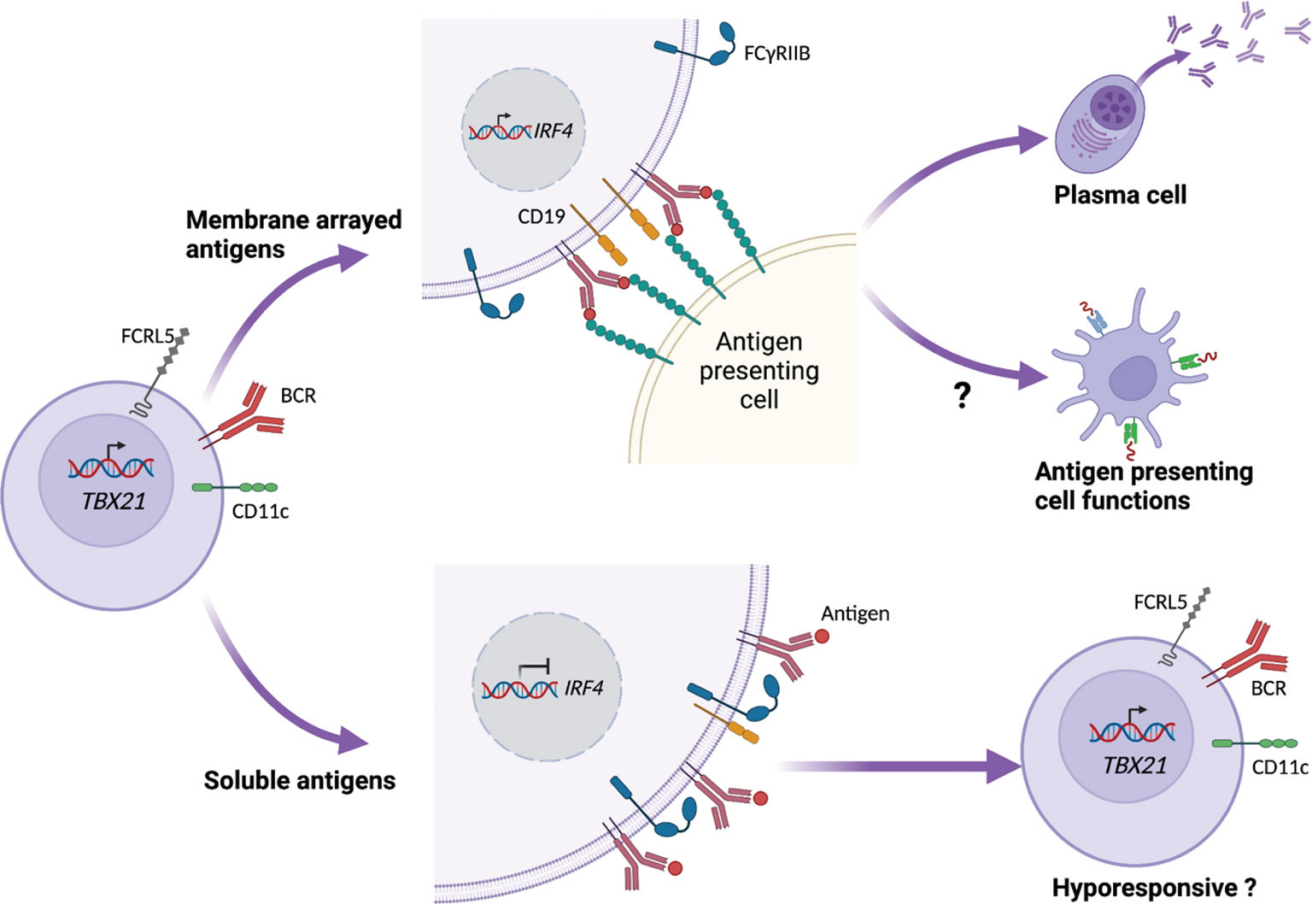
CD21^lo^ B cell responsiveness is dependent on how antigen is presented. CD21^lo^ B cells expressing CD11c, T-bet (*TBX21*), and FCRL5 also express increased levels of inhibitory receptors, such as FcγRIIB. In the context of B cell receptor (BCR) ligation, FcγRIIB reduces the engagement of CD19 with the BCR, thus preventing down stream signaling, leading to hyporesponsiveness. In contrast, CD21^lo^ B cells binding to membrane arrayed antigens establish an immunological synaps that excludes FcγRIIB. This allows CD19 to engage the BCR and promote downstream signaling, leading to transcription of *IRF4* that in turn can drive differentiation to antibody secreting cells. The membrane-arrayed antigens also lead to improved antigen-uptake and could potentially enhance the B cell antigen-presenting cell functions.

A recent study by Ambegaonkar et al. indicates a mechanism of how CD21^lo^CD27^lo^ B cells can come across as dysfunctional in restimulation assays, and as important contributors to the secreted antibody pool in other studies (84). They show that the high expression of inhibitory receptors, and especially FcγRIIB, restricts CD21^lo^CD27^lo^ B cells in their response to soluble antigen. However, in conditions where the BCR ligand or antigen is presented to the cells while fixed in a lipid bilayer, FcγRIIB is excluded from the immunological synapse, allowing the engagement of CD19 with the BCR (84). Such conditions, summarized in **Figure 2**, induce a strong BCR signal leading to the expression of IRF4, which is associated with ASC differentiation (85).

Experiments in mice have indicated that CD11c^+^T-bet^+^ B cells are associated with protection from chronic viral infection (86). However, this effect was not only associated with antibody production but also with other cell-mediated mechanisms. In addition to their potential role as ASC precursors, CD21^lo^ B cells upregulate proteins with important functions in the T-B synapse, such as MHC class II and the co-stimulatory molecules CD80, CD86, OX40, and ICOS-L (78, 80). They are also more efficient than naïve and conventional memory B cells in taking up antigens (84). Together this could indicate that the cells have become more potent as APCs. Indeed, CD11c^+^ B cells in mice were able to improve CD4^+^ T cell activation and proliferation compared with follicular B cells (87, 88) and depletion of CD11c^+^ B cells in mice led to reduced Tfh cell levels (88). In contrast, sorted human CD21^lo^ B cell subsets provided similar CD4^+^ T cell activation as CD21^+^ B cell subsets in an *in vitro* mixed-lymphocyte reaction assay (89). However, since CD11c^+^ and T-bet^+^ B cells also upregulate homing receptors, such as CXCR3, they can migrate to sites of inflammation and provide localized APC functions or potentially complete differentiation to ASCs. Such an effect was recently described by MacLean et al. where they showed that CXCR3^+^ lung-resident memory B cells were recruited to infected foci in the lung in an IFN-γ dependent manner upon reinfection of mice with influenza virus. The cells then differentiated to ASCs at the foci to provide localized antibody production (90).

Overall, these studies highlight that CD21^lo^ CD11c^+^ or T-bet^+^ B cells should no longer be considered as an exhausted or dysfunctional B cell subset, but rather as fully capable of responding to specific signals. Although the extent of the role they play in a systemic immune response to infection such as malaria or during autoimmune disease remains largely unclear, recent studies indicate important functions that need to be further explored.

## Localization and Homing of CD21^lo^ B Cells

One of the many unanswered questions regarding CD21^lo^T-bet^+^ B cells is the localization and homing of such cells. A recent study by Johnson et al., investigating T-bet^+^ B cells in patients undergoing surgery and in mice, showed a similar pattern of distribution in different tissues (75). They also showed that influenza-specific T-bet^+^ B cells were differentially distributed in the spleen, peripheral blood, bone marrow, and lung, indicating that the cells had a preferred tissue homing associated with the infection.

Interestingly, only B cells expressing low levels of T-bet were present in the lymphoid circulation, while T-bet^hi^ B cells were absent from lymph nodes (75). This is consistent with previous studies (26, 91), where B cells lacking CD27 and expressing FCRL4 or FCRL3 were present in lymph nodes, tonsil, and payer patches, but few, if any cells expressing high levels of T-bet (91). Similarly, after influenza vaccination in humans, B cells expressing *TBX21, FCRL5*, and *ITGAX* were present in peripheral blood, but not in the draining lymph nodes (92). This partial or absent expression of markers associated with CD11c^+^T-bet^+^ B cells could indicate that differentiation concurrently changes the receptor expression to promote migration from secondary lymphoid organs to tissues, consistent with low levels of CD62L, CXCR5, and CCR7 on these cells in the blood (32, 73). It is however interesting to note that CD11c^+^ T-bet^+^ B cells are present in the spleen but not in lymph nodes (75). In mice, it was recently described by Song et al, that CD11c^+^T-bet^+^ B cells generated after virus infection were retained in the splenic marginal zone through interactions by LFA-1 and VLA-4, indicating a potential mechanism of splenic retention (68), while the mechanisms of homing to and retention in other tissues remain to be explored. Further studies are also needed to understand how differentiation is associated with migration.

## Conclusion

Upon immune activation, B cells differentiate to provide the host with several important effector functions. Of these, antibody production is without a doubt the most well described, but B cells also provide important roles as APCs in addition to secret different cytokines to both promote and suppress inflammatory responses (15, 93).

Over the last decade, a phenotypically distinct, although heterogenous B cell subset, identified through reduced expression of CD21 and CD27 and upregulation of one or several of T-bet, CD11c, and FcRL4/5, has gained increasing attention. In this review, we have presented recent data generated in different research fields from human samples and mice, and although the inconsistent use of names and markers to identify these cells often makes direct comparisons difficult, several studies point toward these cells having largely overlapping phenotypic and transcriptional signatures and homing patterns. However, many studies also point to substantial heterogeneity in the markers expressed by these cells between diseases and over time and also between mice and humans. This illustrates that further studies that directly compare the cells between diseases, time-points, tissues, organisms, or stimulations using the same systematic approach are needed. Such comparative studies would also be very useful for the research community to decide on a more systematic nomenclature for these cells.

## Author Contributions

All listed authors have made a substantial, direct and intellectual contribution to the work, and approved it for publication. ADCG and LK contributed equally to the work and the author order was determined by dice.

## Funding

This work was supported by grants from the Swedish Research Council (2019-01940), Åke Wiberg foundation (M18-0076), Magnus Bergvall Foundation (2017-02043 and 2018-02656), Tore Nilsson Foundation (2018-00608), and Karolinska Institutet Faculty funds (2020-00878) to CS. None of the sources of funding has an interest in the subject matter or materials discussed in the submitted manuscript. ADCG is supported by Karolinska Institutet PhD program faculty funds.

## Conflict of Interest

The authors declare that the research was conducted in the absence of any commercial or financial relationships that could be construed as a potential conflict of interest.

## Publisher’s Note

All claims expressed in this article are solely those of the authors and do not necessarily represent those of their affiliated organizations, or those of the publisher, the editors and the reviewers. Any product that may be evaluated in this article, or claim that may be made by its manufacturer, is not guaranteed or endorsed by the publisher.
